# Exercise as Prescription Therapy: Benefits in Cancer and Hypertensive Patients

**Published:** 2014-12-19

**Authors:** Laura Stefani, Nicola Maffulli, Gabriele Mascherini, Lorenzo Francini, Cristian Petri, Giorgio Galanti

**Affiliations:** 1 Sports Medicine Center, Florence, Italy.; 2 Department of Musculoskeletal Disorders, Faculty of Medicine and Surgery,University of Salerno, Salerno, Italy; and Queen Mary University of London, Barts and The London School of Medicine and Dentistry, Institute of Health Sciences Education, Centre for Sports and Exercise, UK

**Keywords:** exercise therapy, life style, chronic disease, body composition

## Abstract

PURPOSE Exercise therapy in patients with metabolic chronic disease produces several positive response. This study aims to verify the effects of fast walking associated to a resistance exercise to reduce cardiovascular risk factor. METHODS: Two groups of subjects (10 cancer survivors and 19 hypertensive patients) were evaluated by 6-Minute Walking Test (6MWT), bioimpedance, the Sit & Reach Test (S&R) evaluate the flexibility, Handgrip and 30” Chair Test for muscular strength. Patients were tested before and after 3 months of regular physical exercise. RESULTS: A significant change in anthropometric parameters was observed (BMI: T0 = 29.2±6.8, T3= 27.4±4.4 kg/m2 p<.001; waist circumference: T0=92.5±14.1, T3=92.1±12.8 cm, p<.05) in the hypertensive population. A predominant improvement of the cardiovascular parameters was observed in the cancer survivors (rest DBP T0=76.4±6.5, T3=72.2±7.1 mmhg p<.05; 6MWT: T0=487.8±116.0, T3= 525.6±117.3 m p<.05; S&R: T0= 0.4±7.4, T3=4.1±6.1 cm p<.05). CONCLUSION: A combined aerobic and resistance exercise programme can improve cardiovascular risk factors in hypertensive subjects. The same programme induces improvement in exercise tolerance and flexibility variables in cancer survivors.

## INTRODUCTION:

Marked technological and pharmacological advances in the management of cardiovascular disease and its associated risk factors have been made [1]. The positive effects of exercise as a prescription therapy, especially fast walking programme, in patients with chronic metabolic disease have been recently demonstrated [2]. Hypertension remains a major public health problem [3]. Cancer is the second cause of death, and is superimposed to metabolic conditions including obesity or overweight. Non supervised exercise programmes have been proposed in both cancer and hypertensive patients [4]. Many clinical trials in this field are limited to a few weeks, and do not investigate the effects of the combination of the aerobic and resistance exercise. In addition, the global response and the possible differences between these two conditions after a short period of training have not been investigated. The present study aims to verify the possible impact of a non supervised aerobic exercise programme associated to resistance exercise on several anthropometric and haemodynamic variables in cancer and hypertensive patients.

## METHODS:

Two groups of subjects (one composed of 5 female breast and 5 male colon overweight cancer survivors, aged 48.8 ±4.6, in good clinical conditions, previously treated with chemotherapy, without evidence of cardiovascular disease; the other of 19 subjects, aged 54.3± 3.7 affected by mild hypertension under pharmacological management) took part in the present study. They were prescribed an exercise programme composed of aerobic (fast walking) and resistance exercises (home based exercises for the upper and lower limb, without weight lifting at the beginning of the programme). Lifestyle was evaluated by a questionnaire to ascertain their levels of physical activity. Following the ACSM guidelines, the intensity of aerobic exercise was set at 60% maximal effort for at least three times a week for at least three months The resistance exercises programme included simple exercises: home based isotonic exercise for the upper and lower limb, without weight lifting at the beginning of the program. Initially, we recommended 2 sets of 10 repetitions per each set to increase according to subjective perception, never exceeding 3 sets of 20 repetitions for each exercise. Flexibility exercises involving the calf muscles, quadriceps and hamstring consisted of one set of static stretching exercises in which the subject maintained the stretch for at least one minute.

The aerobic exercise prescription was based on the results of the 6-Minute Walking Test (6MWT): heart rate, respiratory rate, systolic and diastolic blood pressure (DBP) were assessed at rest and at the end of the test [5]. The intensity of the aerobic exercise was obtained by the formula (HRt = 60% × (HRmax − HRrest) + HRrest,) where HRt is the target heart rate, HRmax is maximum heart rate, and HRrest is heart rate at rest. Heart rate values were evaluated using a dedicated software (Zephyr Bioharness model 9600.0091, Annapolis, Maryland U.S.A.) for telemetry using an elastic strap placed on the chest. The heart rate corresponding to 60% of the final value obtained was the target of the intensity of the exercise corresponding to the maximum tolerated effort per each subject. Following the ACSM guidelines, the duration of the aerobic exercise per session was 30 minute for at least 3 times a week, up to a maximum of 150 min per week. The duration of the aerobic exercise, was established also from the effective effort tolerance derived from the CR10 scale evaluation at the end of the 6MWT test. The results of the 6MWT were integrated with the CR10 score to determine the final intensity of the aerobic exercise in term of heart rate per minute [6]

We measured the distribution of the Intra (ICW) and Extracellular (ECW) water by Bioimpedance (BIA) analysis [7] The data collected were processed with a dedicated software (Bodygram pro 3.0). We measured weight, height, Body Mass Index (BMI), Basal Metabolism (BM), Phase Angle (PA), Free Fat Mass (FFM), Fat Mass (FM), Total Body Water (TBW), Extra Cellular Water (ECW), Intra Cellular Water (ICW), Body Cellular Mass (BCM).

For resistance exercise prescription, the muscle strength of the upper and lower limbs and flexibility were evaluated by the Sit & Reach Test (S&R), the Handgrip, and the 30” Chair Test [8]. All the tests were performed at baseline and after 3 month of regular physical exercise.

## RESULTS:

At baseline, both groups were at the upper limits of the overweight range. After 3 months of regular and unsupervised mixed exercises, a significant reduction of anthropometric variables was evident (Tab. 1) Blood Pressure remained unchanged. The cardiovascular and resistance exercises variables improved significantly in the cancer group, but remained unchanged in the hypertensive group (Tab. 1)

## DISCUSSION

The number of cancer survivors is now growing. In parallel, the amount of aged subjects with increased cardiovascular risk is increasing (11,12). The presence of metabolic syndrome in cancer represents often the main reason to introduce new treatment strategies. The onset of metabolic syndrome in cancer survivors may cause the long term morbidities the complications of which can interfere with cancer treatment and worsen the quality of life. Regular physical exercise has antinflammatory effects, which may benefit cancer patients (13). The adipose tissue is the primary site of production of inflammatory factors, as IL6, TNF, and it can be responsible, in the tumorgenesis process, for the activation of distant metastases (14). Aerobic [9] and resistance exercise [10] can each produce significant positive effects on body fitness, muscle function and body composition, both in hypertensive patients and in cancer survivors. The combination of aerobic and resistance exercise can reduce the metabolic risk factors in cancer survivors and in hypertensive patients. The present study has shown that in cancer survivors a short period of mixed aerobic and resistance exercises, a predominant improvement in aerobic exercise tolerance and flexibility. This is not the case in hypertensive patients, in whom it is possible that the duration and intensity of the exercise programme used in the present study is not sufficient to elicit the desired effect.

These results support the short term efficacy of non supervised and combined aerobic and resistance exercise programme. The effects of such exercise programme are different in these two groups of patients, and will need further investigations with larger cohorts and longer follow up. Nevertheless, the results are promising, and may result in a greater rate of exercise prescription in these patients, with reduction of cardiovascular risk factors and improvement in quality of life.

## Figures and Tables

**Figure:**
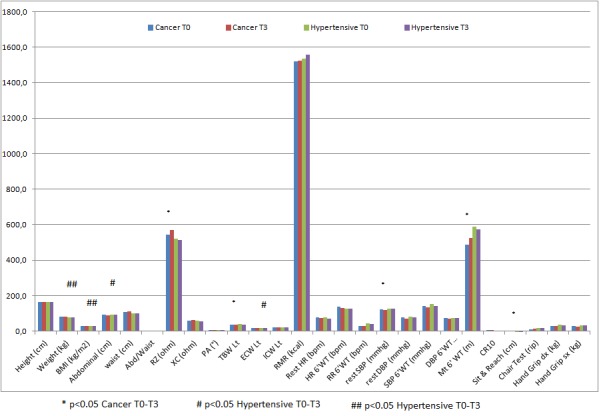


**Table 1:** results of 3 months of exercise in all parameters evaluated

	Cancer T0	Cancer T3	p value	Hypertensive T0	Hypertensive T3	p value
Age (years)	48.8 ± 4.6			54.3±3.7		
Height (cm)	165.5±12.0	165.5±12.0	NS	163.5±10.0	163.5±10.0	NS
Weight (kg)	82.2±23.5	80.6±21.6	NS	77.9±16.6	76.2±15.8	<0.00
BMI (kg/m2)	30.3±10.3	29.8±9.7	NS	29.2±6.8	27.4±4.4	<0.00
Abdominal (cm)	92.4±19.2	90.9±17.3	NS	92.5±14.1	92.1±12.8	<0.05
waist (cm)	109.2±21.1	110.5±19.2	NS	101.1±8.6	101.0±8.5	NS
Abd/Waist	0.8±0.1	0.8±0.1	NS	0.9±0.1	0.9±0.1	NS
RZ (ohm)	542.7±91.2	569.7±104.1	<0.05	520.5±82.8	513.4±86.0	NS
XC (ohm)	59.9±10.5	61.4±11.6	NS	58.1±17.6	55.9±12.3	NS
PA (°)	6.4±1.1	6.2±0.7	NS	6.4±1.4	6.2±1.1	NS
TBW (Lt)	38.1±8.2	37.0±7.9	<0.04	39.9±8.6	38.1±8.1	NS
ECW (Lt)	16.9±3.9	16.7±3.8	NS	17.5±3.7	17.2±3.9	<0.05
ICW (Lt)	21.2±4.9	20.3±4.6	NS	20.9±4.8	21.9±4.2	NS
BMR (kcal)	1521.1±152.8	1525.0±182.7	NS	1536.4±187.5	1556.1±174.5	NS
Rest HR (bpm)	77.1±9.2	74.0±12.4	NS	76.3±12.1	71.2±9.9	NS
HR 6’WT (bpm)	137.4±12.9	131.5±15.2	NS	126.9±18.2	127.9±15.3	NS
RR 6’WT (bpm)	30.0±6.7	29.0±5.3	NS	42.5±19.6	39.5±9.1	NS
rest SBP (mmhg)	121.7±8.7	118.3±13.2	NS	128.3±7.5	126.9±12.5	NS
rest DBP (mmhg)	76.4±6.5	72.2±7.1	<0.05	82.0±7.1	78.1±9.9	NS
SBP 6’WT (mmhg)	141.7±10.9	135.3±7.1	NS	153.8±8.3	141.8±12.0	NS
DBP 6’WT (mmhg)	73.7±6.3	70.6±6.8	NS	75.6±8.6	72.5±6.4	NS
6’M WT (m)	487.8±116.0	525.6±117.3	<0.05	590.0±67.3	575.4±56.9	NS
CR10	5.7±3.3	4.9±2.9	NS	3.0±2.1	2.7±2.1	NS
Sit & Reach (cm)	0.4±7.4	4.1±6.1	<0.05	−5.2±8.3	−6.2±6.5	NS
Chair Test (rip)	11.8±2.2	14.1±3.9	NS	16.6±3.4	16.4±2.8	NS
Hand Grip right (kg)	29.3±17.2	27.5±5.1	NS	36.3±10.2	33.8±11.6	NS
Hand Grip left (kg)	30.5±13.4	27.2±5.6	NS	34.0±9.1	33.2±11.9	NS

Legend: BMI (Body Mass Index); Abd/Waist (Abdominal/waist); RZ (Resistance); XC(Reactance); PA (Phase Angle); TBW (Total Body Water); ECW (Extra Cellular Water); ICW (Intra Cellular Water); BMR (Body Metabolic Rate); HR(Heart Rate); HR6’WT(Heart Rate 6 minute Walking Test); RR 6’WT (Respiratory Rate 6 minute Walking Test); SBP(Systolic Blood Pressure); DBP (Diastolic Blood Pressure); CR10(Category-Ratio scale of Effort),
